# Value of a short non-contrast CMR protocol in MINOCA

**DOI:** 10.1007/s00330-023-10096-2

**Published:** 2023-08-15

**Authors:** Marco Gatti, Anna Palmisano, Mattia Gerboni, Riccardo Cau, Alessandra Pintus, Michele Porcu, Davide Tore, Davide Vignale, Alessandro Andreis, Laura Bergamasco, Gaetano Maria De Ferrari, Antonio Esposito, Luca Saba, Paolo Fonio, Riccardo Faletti

**Affiliations:** 1 Radiology Unit, Department of Surgical Sciences, University of Turin, Via Genova 3, 10126 Turin, Italy; 2 Clinical and Experimental Radiology Unit, Experimental Imaging Center, IRCCS San Raffaele Scientific Institute, Via Olgettina 60, 20132 Milan, Italy; 3 School of Medicine, Vita-Salute San Raffaele University, Milan, Italy; 4 Department of Radiology, AOU Cagliari, University of Cagliari, Cagliari, Italy; 5 Division of Cardiology, Department Cardiovascular and Thoracic, Città Della Salute E Della Scienza Hospital, University of Turin, Turin, Italy

**Keywords:** Chest pain, MINOCA, Myocarditis, Takotsubo cardiomyopathy, Myocardial infarction

## Abstract

**Objectives:**

To evaluate the diagnostic performance of a short non-contrast CMR (ShtCMR) protocol relative to a matched standard comprehensive CMR (StdCMR) protocol in patients with myocardial infarction with non-obstructive coronary arteries (MINOCA).

**Methods:**

This multicenter retrospective study included patients with a working diagnosis of MINOCA who underwent a StdCMR between January 2019 and December 2020. An expert and a non-expert reader performed a blinded reading with the ShtCMR (long-axis cine images, T2w-STIR, T1- and T2-mapping). A consensus reading of the StdCMR (reference standard) was performed at least 3 months after the ShtCMR reading session. Readers were asked to report the following: (1) diagnosis; (2) level of confidence in their diagnosis with the ShtCMR; (3) number of myocardial segments involved, and (4) functional parameters.

**Results:**

A total of 179 patients were enrolled. The ShtCMR lasted 21 ± 9 min and the StdCMR 45 ± 11 min (*p* < 0.0001). ShtCMR allowed reaching the same diagnosis as StdCMR in 85% of patients when interpreted by expert readers (rising from 66% for poor confidence to 99% for good, *p* = 0.0001) and in 73% (*p* = 0.01) by non-expert ones (60% for poor vs 89% for good confidence, *p* = 0.0001). Overall, the ShtCMR overestimated the ejection fraction, underestimated cardiac volumes (*p* < 0.01), and underestimated the number of segments involved by pathology (*p* = 0.0008) when compared with the StdCMR.

**Conclusion:**

The ShtCMR was found to be a debatable alternative to the StdCMR in patients with MINOCA. Nevertheless, when an experienced reader reaches a good or very good diagnostic confidence using the ShtCMR, the reader may choose to stop the examination, reducing the length of the CMR without affecting the patient’s diagnosis.

**Clinical relevance statement:**

A short non-contrast CMR protocol may be a viable alternative to standard protocols in selected CMR studies of patients with MINOCA, allowing for faster diagnosis while reducing time and resources and increasing the number of patients who can be scanned.

**Key Points:**

• *The ShtCMR lasted 21* ± *9 min and the StdCMR 45* ± *11 min (p* < *0.0001).*

• *In 57% of patients with MINOCA, the experienced reader considers that contrast medium is probably not necessary for diagnosis without affecting the patient’s diagnosis (99% of agreement rate between ShtCMR and StdCMR).*

## Introduction

Acute coronary syndrome (ACS) is a leading cause of mortality, with coronary artery disease representing its most common origin [1, 2]. However, it is estimated that 7 to 15% of patients presenting with an ACS have myocardial damage but no obstructed coronary arteries or other clinically evident reasons that might justify the acute presentation. To describe such clinical scenario, the term myocardial infarction with non-obstructive coronary arteries (MINOCA) was established [3, 4].

According to the European Society of Cardiology (ESC) guidelines and American College of Cardiology/American Heart Association (ACC/AHA) Joint Committee [3–5], cardiac magnetic resonance (CMR) is a key diagnostic technique in the algorithm for the differential diagnosis of patients with MINOCA for its ability to characterize the myocardium in addition to the morphofunctional evaluation. CMR was found to detect the underlying etiology in 74–87% of cases [6–9] and to change the clinical diagnosis in around 50% of patients [6]. CMR should, however, be performed within few days of symptom onset [9], since a delayed evaluation may result in a false-negative evaluation for edema resolution [10] and a delayed diagnosis may have a negative impact on the patient’s prognosis [7]. Unfortunately, CMR is not widely practiced, particularly in emergency settings, since it needs a lengthy acquisition time and requires the administration of contrast medium, which is not always desirable.

In recent years, the application of new CMR techniques, such as mapping sequences, revolutionized the CMR scenario since they can characterize the myocardium in an accurate and quantitative mode. These sequences provide a wide range of additional information, from edema to fibrosis, that opens up the possibility of a gadolinium-free protocol combining T2-based imaging with T1 mapping [11–15]. A protocol requiring reduced acquisition time with comparable diagnostic accuracy as standard contrast-enhanced CMR is highly desirable to speed up the diagnostic work-up in MINOCA patients, especially when the administration of contrast agents is contraindicated (i.e., patients with renal insufficiency or specific allergies).

To the best of our knowledge, the diagnostic value of a short non-contrast CMR (ShtCMR) protocol in patients with MINOCA has not yet been investigated. Hence, we planned a retrospective study aimed at evaluating how the diagnostic performance of a ShtCMR protocol lasting around 20 min without contrast agent compared with that of a matched standard comprehensive CMR (StdCMR) protocol on patients admitted to the emergency department for MINOCA.

## Materials and methods

### Study design

This retrospective study analyzed all CMR studies of consecutive adult patients (age 18–80 years) admitted between January 2019 and December 2020 in three tertiary care university hospitals with a working diagnosis of MINOCA. The study was approved by the institutional review boards. Before CMR, all patients herein considered were informed about the possible use of their data for study purposes and gave their consent. Data were anonymized prior to analysis.

**Fig. 1:**
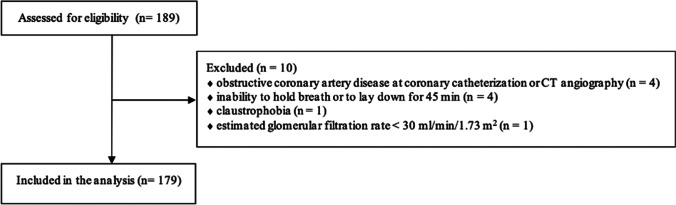
Enrollment flowchart

To enter the study population, inclusion criteria were as follows: (i) acute chest pain or anginal equivalents; (ii) myocardial injury markers (elevated troponin T/troponin I), and a (iii) comprehensive CMR scan protocol with contrast media acquired within 14 days from the symptom’s onset. Exclusion criteria were as follows: (i) obstructive coronary artery disease at coronary catheterization or CT angiography; (ii) inability to hold breath or to lie down for 45 min; (iii) claustrophobia; (iv) recent history of alimentary/alcoholic/respiratory intoxication; (v) estimated glomerular filtration rate < 30 mL/min/1.73 m^2^; (vi) history of allergic reaction to MR contrast media; and (vii) pregnancy or breastfeeding. The enrollment flowchart is shown in Fig. 1.

The primary goal of the study was to compare the diagnostic performance of ShtCMR with that of StdCMR, when analyzed by an experienced or a less experienced reader. The secondary goal was to test the ability of ShtCMR in assessing disease extent and left ventricular (LV) functional parameters.

### CMR protocol

CMR imaging was performed with a 1.5-T scanner (Achieva and Achieva dStream, Philips) using a body coil (32-channel) according to a standardized protocol [16, 17].

All patients underwent a StdCMR. For functional analysis, cine steady-state free precession (cine-SSFP) CMR images were acquired in four-, two-, and three-chamber and short-axis views. Edema-sensitive black-blood T2-weighted short tau inversion recovery (T2w-STIR) images were acquired on a long- and a short-axis chamber covering the entire LV. Late gadolinium enhancement (LGE) images were acquired 10 min after contrast administration using segmented inversion recovery gradient echo sequences (IR-GRE) on a long- and a short-axis chamber covering the entire LV. Moreover, for every patient, T1 mapping (pre- and post-contrast) with a modified Look-Locker inversion recovery (MOLLI) technique and T2 mapping with an optimized gradient and spin echo sequence (GraSE) were performed.

### Imaging analysis

All CMR studies were evaluated using a dedicated software (IntelliSpace Portal 8.0, Philips Healthcare). For StdCMR, the reader had available all the acquired sequences, whereas for ShtCMR, they were “limited” to long-axis cine (2-, 3-, and 4-chamber view), T2w-STIR (long- and short-axis view), and T1 and T2 mapping (short-axis view).

In each center, CMR scans were interpreted by two readers: a radiologist with > 5 years of cardiac imaging experience (A.P., M.G., R.C.) and a resident (D.T., M.G., A.P.) with roughly 100 CMR cases interpreted. Each reader analyzed first the ShtCMR images in a blinded reading and, after at least 3 months, the StdCMR images. The arrangement of the sessions was chosen to prevent bias in the StdCMR reading due to uncontrollable visual memories. Readers were asked to report the following data in the ShtCMR analysis:

1. Final diagnosis (i.e., acute myocarditis, myocardial infarction, takotsubo cardiomyopathy, negative or others)
2. The degree of confidence felt in the diagnosis with ShtCMR expressed as need for contrast medium administration on a likelihood Likert scale: (1) definitely yes, (2) probably yes, (3) possibly, (4) probably not, and (5) definitely not, as self-estimated by the reader on the likelihood Likert scale (“good” confidence corresponding to LS ≥ 4)
3. Number of myocardial segments involved
4. LV end-diastolic and systolic volume (LVEDV and LVESV) and the ejection fraction (LVEF) using the biplane long-axis technique [18, 19]

### Statistical analysis

Continuous variables, expressed as average ± standard deviation, were compared with Mann–Whitney’s test for two independent distributions or Wilcoxon’s test for two matched distributions. Categorical variables were compared with Fisher’s exact test when independent or McNemar’s test when matched. The concordance over the 5 possible diagnoses was evaluated with Cohen’s kappa coefficient.

The receiving operating characteristics (ROC) curve and its accuracy metrics were used to evaluate the diagnostic performance of ShtCMR against StdCMR and to dichotomize continuous variables recognized significant by Mann–Whitney’s test.

Significant association corresponded to *p* < 0.05. Analyses were performed using Statplus for Macintosh Build 8.0.1.0/Core v7.7.11, 2021 (AnalystSoft).

## Results

One hundred seventy-nine MINOCA patients constituted the final study cohort. Table 1 presents the population characteristics and relative CMR findings. CMRs were performed 5 ± 4 days after symptom onset (troponin T peak 1651 ± 2368 ng/mL, normal range < 14 ng/mL), leading to the diagnosis of 95 (55.3%) acute myocarditis, 35 (19.6%) myocardial infarction, 29 (16.2%) takotsubo cardiomyopathy, 3 (1.7%) others, and 13 (7.3%) negative exams.

**Table 1:** Clinical and cardiovascular magnetic resonance functional characteristic of enrolled patients

Characteristics	Value
Sample size	179
Female (%)	72 (40%)
Age (years)	48 ± 20
Body surface area (m^2^)	1.83 ± 0.22
Troponin T peak value (ng/mL)	1651 ± 2368
Creatine kinase (U/L)	364 ± 772
LVEDV (mL)	137 ± 44
LVESV (mL)	61 ± 34
LVEDVi (mL/mq)	76 ± 20
LVESVi (mL/mq)	34 ± 16
LVEF (%)	57 ± 11
LV mass (g)	94 ± 30
LV mass index (g/mq)	52 ± 14
RVEDV (mL)	142 ± 46
RVESV (mL)	64 ± 30
RVEDVi (mL/mq)	77 ± 21
RVESVi (mL/mq)	35 ± 15
RVEF (%)	56 ± 8

*LVEDV* left ventricular end diastolic volume, *LVESV* left ventricular end systolic volume, *LVEDVi* left ventricular end diastolic volume index, *LVESVi* left ventricular end diastolic volume index, *LVEF* left ventricular ejection fraction, *RVEDV* right ventricular end diastolic volume, *RVESV* right ventricular end systolic volume, *RVEDVi* right ventricular end diastolic volume index, *RVESVi* right ventricular end diastolic volume index, *RVEF* right ventricular ejection fraction

### Comparison of short CMR protocol diagnosis with matched standard CMR protocol diagnosis

The five possible diagnoses were as follows: acute myocarditis, myocardial infarction, takotsubo cardiomyopathy, negative, or others. The comparison between diagnoses reached with ShtCMR and the matched ones with StdCMR on the 179 cases was carried out in three independent ways, also distinguishing between expert and non-expert reader, and for each of them, good vs poor degree of confidence.

**Fig. 2:**
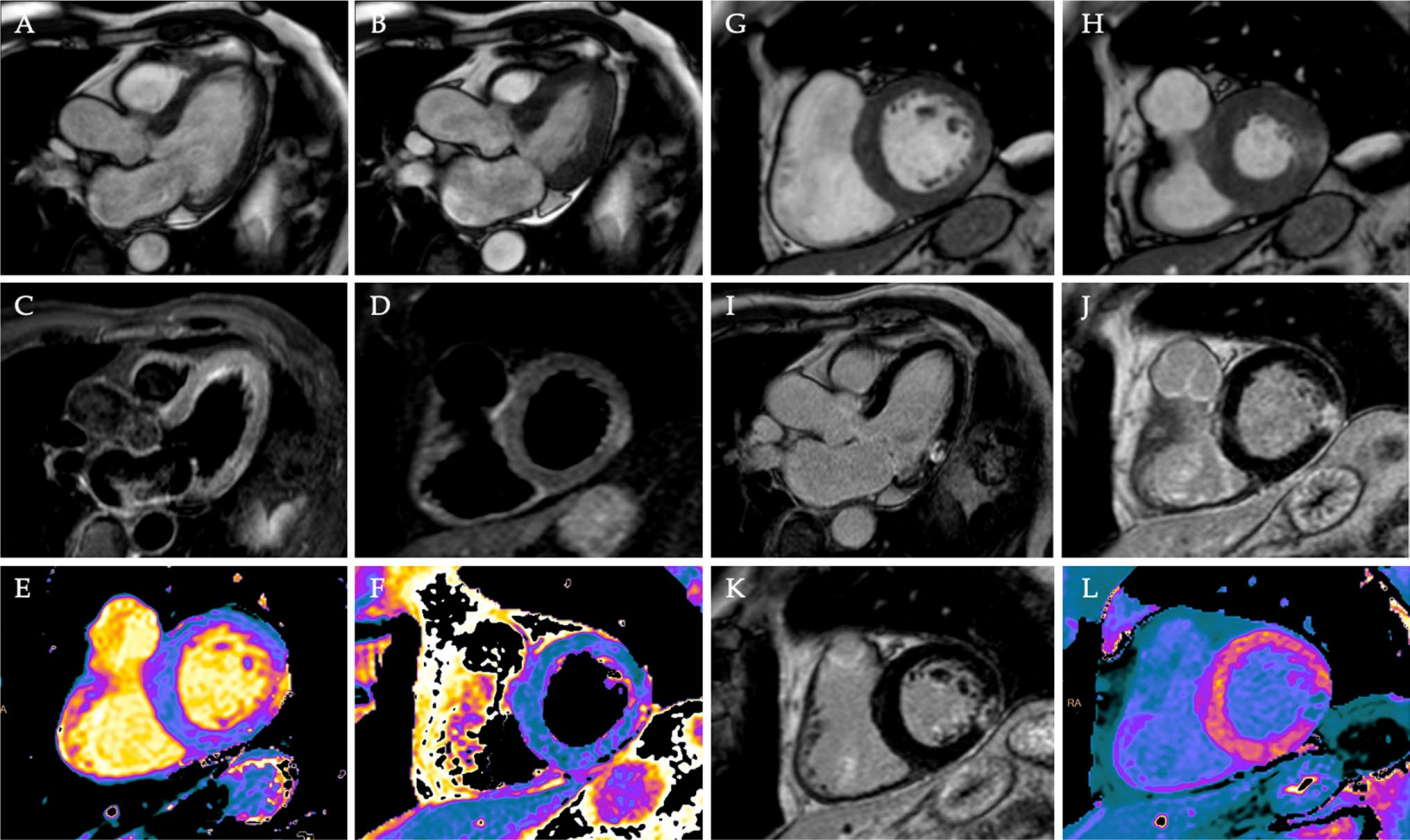
A 67-year-old male patient was admitted to the emergency department with acute chest pain, elevated troponin (troponin T 21 ≥ 280 ng/mL, normal value < 34 ng/mL), and non-ST elevation on the electrocardiogram. In the absence of significant stenosis, invasive coronary angiography revealed 50% stenosis of the anterior descending artery, 50% stenosis of a secondary branch of the first diagonal branch, and irregularities of the right and circumflex coronary arteries. CMR was performed 3 days later. Images from the short protocol are shown on the left panel (**A**, 3ch-view diastolic frame; **B**, 3ch-view systolic frame; **C** and **D**, 3ch and short-axis short tau inversion recovery (STIR) image; **E**, T1 native map; and **F**, T2 map), and contrast-enhanced cine-SSFP (**G**–**H**), late gadolinium enhancement (LGE) sequences (**I**–**K**), and postcontrast T1 map (**L**) are shown on the right. On the short protocol, CMR systolic function was normal (EF: 56%), with focal areas of hypokinesis in the inferolateral segment (**A**, **B**). A focal area of subepicardial edema in the infero-lateral wall is associated with increased native T1 (**E**) and T2 (**F**) values. As a result, the expert reader’s short protocol diagnosis was myocarditis albeit with a score of 4 for the presence of hypokinesia. However, post-contrast cine-SSFP images revealed a slight transmural enhancement in the infero-lateral wall, as well as a small area of hypointensity, indicating microvascular obstruction. These findings were clearly visible on 3ch-view LGE (**I**), 2ch-view LGE (**J**, **K**), and post-contrast T1 mapping (**L**), demonstrating a transmural scar associated with the no-reflow phenomenon. As a result, acute myocardial infarction without obstructive coronary arteries was the correct diagnosis. The presence of subendocardial myocardial hemorrhage on STIR images was responsible for a misdiagnosis in the short non-contrast protocol

Table 2 reports the number and percentage of equal diagnoses (index of agreement, IoA). Overall, IoA increases significantly with the reader’s experience (*p* = 0.001) and level of confidence (*p* < 0.0001). Specific results are given for acute myocarditis, myocardial infarction, and takotsubo cardiomyopathy. The diagnosis of myocardial infarction without contrast medium was the most challenging for both expert and non-expert readers. Indeed, the only case of discordant diagnosis by an expert reader with high confidence in ShtCMR concerned a 67-year-old male patient admitted to the emergency department with acute chest pain, elevated troponin (troponin T 21 ≥ 280 ng/mL, normal value < 34 ng/mL), and no ST elevation on the electrocardiogram, which was labeled as acute myocarditis with ShtCMR and as myocardial infarction with StdCMR. Figure 2 shows images from the patient’s ShtCMR and StdCMR.

**Table 2:** Agreement of ShtCMR diagnosis with StdCMR diagnosis (benchmark) by readers with different experiences and levels of self-estimated confidence

		Expert reader	Non-expert reader	*p*
All cases (*N* = 179)	All 179	152/179 (85%)	130/179 (73%)	*0.001*
	[*N (LS* ≥ *4*)]	102	79	*0.02*
	Good confidence	101/102 (99%)	70/79 (89%)	*0.003*
	Poor confidence	51/77 (66%)	60/100 (60%)	0.43
	*p*	< *0.0001*	< *0.0001*	
Acute myocarditis (*N* = 99)	All 99	86/99 (87%)	83/99 (84%)	0.25
	[*N (LS* ≥ *4*)]	57	42	0.05
	Good confidence	57*/57* (100%)	38/42 (90%)	*0.03*
	Poor confidence	29/42 (69%)	45/57 (79%)	0.38
	*p*	< *0.0001*	0.21	
Myocardial infarction (*N* = 35)	All 35	27/35 (77%)	15/35 (43%)	*0.001*
	[*N (LS* ≥ *4*)]	22	10	*0.008*
	Good confidence	21*/22*(95%)	9/10 (90%)	> 0.99
	Poor confidence	6/13 (46%)	6/25 (24%)	0.19
	*p*	*0.002*	*0.0006*	
Takotsubo cardiomyopathy (*N* = 29)	All 29	24/29 (83%)	20/29 (69%)	0.13
	[*N (LS* ≥ *4*)]	20	20	> 0.99
	Good confidence	20/20 (100%)	17/20 (85%)	0.11
	Poor confidence	4/9 (44%)	3/9 (33%)	> 0.99
	*p*	*0.001*	*0.01*	

*ShCMR* short protocol, *StdCMR* standard protocol, *LS* likelihood Likert scale

Significant differences were reported in italics

Table 3 reports Cohen’s concordance coefficient *k* over the five possible diagnoses. For both readers, the concordance increased with the reader’s level of confidence, going from moderate (*k* ≅ 0.5) when the diagnosis was performed with poor confidence to good–excellent (*k* > 0.8) when performed with a high confidence.

**Table 3:** Cohen’s concordance coefficient

	Expert reader	Non-expert reader
All cases	*k* = 0.77 (0.52–1)	*k* = 0.61 (0.37–0.86)
Good confidence	*N* = 102 *k* = 0.99 (0.97–1)	*N* = 79 0.83 (0.70–0.95)
Poor confidence	*N* = 77 *k* = 0.55 (0.39–0.71)	*N* = 100 *k* = 0.45 (0.22–0.68)

**Fig. 3:**
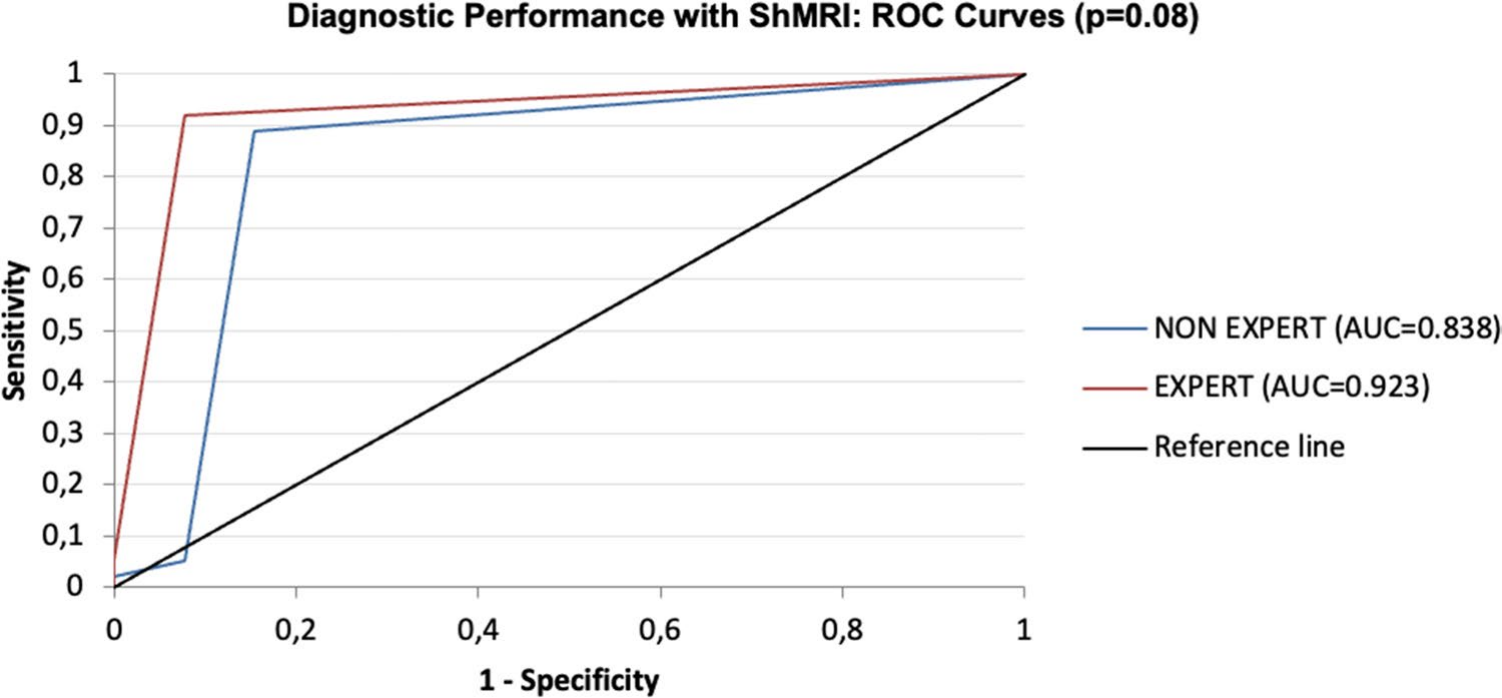
ROC curves for the expert (blue line) and the non-expert reader (red line)

Table 4 and Fig. 3 report the outcome of the ROC curve procedure for assessing the diagnostic performance of ShtCMR using as reference StdCMR. The diagnostic performance of ShtCMR was very good (AUC = 0.92, sensitivity and specificity 92%) with expert readers and good (AUC = 0.84, sensitivity 89%, specificity 84%) for non-expert ones (*p* = 0.08). Better confidence significantly increased performance (*p* < 0.04) for both readers.

**Table 4:** Diagnostic performance of the ShtCMR in respect to the StdCMR

Diagnosis		Expert	Non-expert	*p*
All	AUC	0.92 (0.85–0.99)	0.84 (0.69–0.99)	0.08
	Sensitivity	0.92	0.89	
	Specificity	0.92	0.85	
	Positive predictive value	0.99	0.98	
	Negative predictive value	0.60	0.50	
Good confidence	AUC	1.0 (1–1)	0.96 (0.92–1)	0.08
	Sensitivity	1	0.93	
	Specificity	1	1	
Poor confidence	AUC	0.86 (0.75–0.97)	0.71 (0.41–1)	0.12
	Sensitivity	0.81	0.86	
	Specificity	0.90	0.67	
	*p*	*0.02*	*0.04*	

Significant differences were reported in italics

### Assessment of myocardial injury extension

**Fig. 4:**
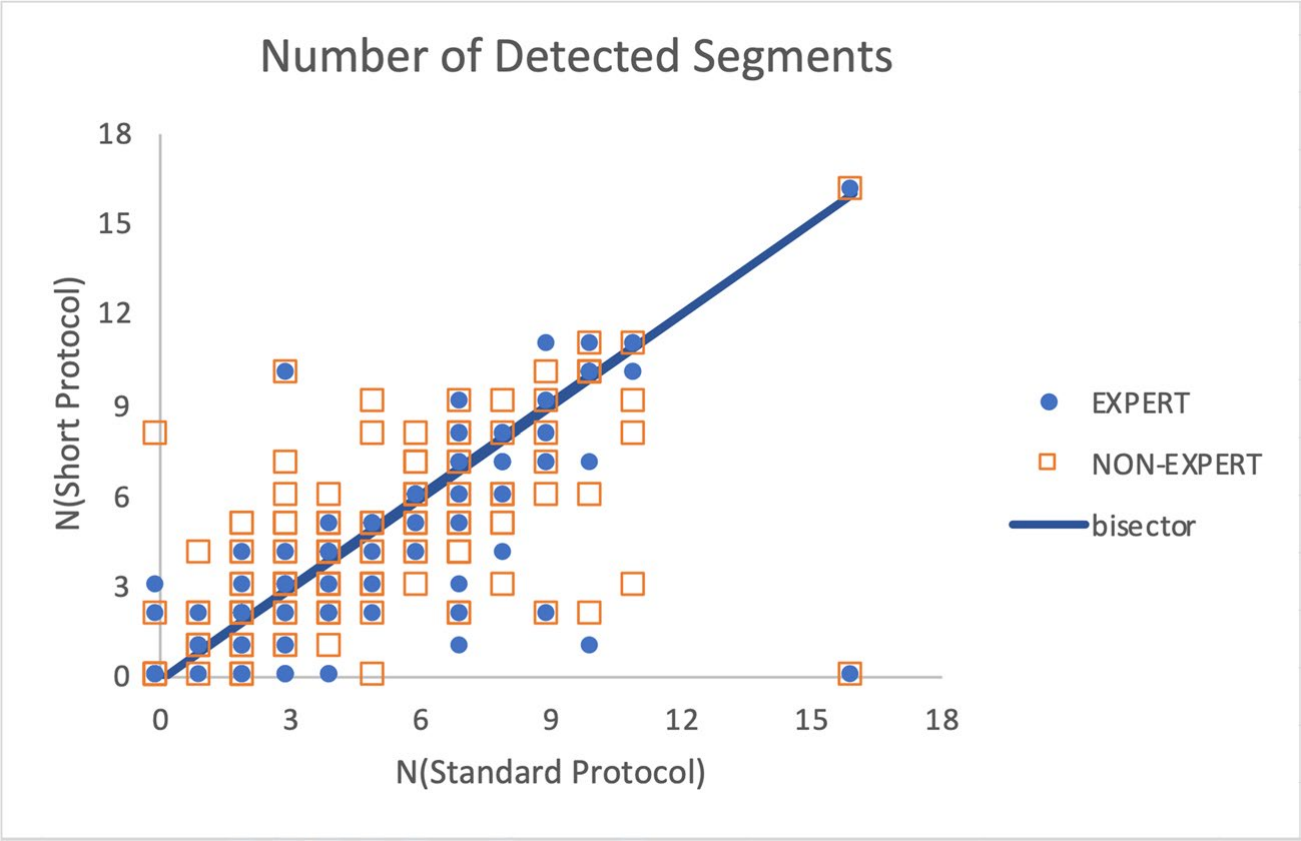
Number of segments detected with short (vertical axis) and standard protocol (horizontal axis by the expert radiologist (blue dots) and the non-expert radiologist (empty red squares). The bisector corresponds to the ideal situation of parity in the number of segments detected with the two protocols

The comparison of the number *N* of segments involved observed with ShtCMR revealed a good agreement between expert and non-expert readers (3.8 ± 3.1 for the former vs 4.0 ± 2.9 for the latter, *p* = 0.77) (Fig. 4). These numbers are, however, significantly (*p* = 0.0008) lower than the value 4.5 ± 3.1 determined with StdCMR.

For the senior reader, the ROC curve identified *N* > 2 as the region significantly associated with good confidence in formulating a diagnosis without the help of the contrast medium (AUC = 0.80, sensitivity 80%, and specificity 70%). Conversely, for the non-expert reader, the number of segments observed was not determinant (AUC = 0.61, barely above chance).

### Assessment of ventricular function and cardiac volumes

Table 5 reports the evaluations of ventricular function and volumes by ShtCMR and by StdCMR. Except for the assessment of the LVEDV and LVEDVi by the experienced reader (*p* = 0.70 and 0.425), ShtCMR significantly underestimates the volumes and overestimates the ejection fraction, with a larger gap for the non-expert reader (*p* < 0.0001).

**Table 5:** Evaluation of ventricular function and cardiac volumes: comparison between reader with short and standard protocol within observer of difference experiences

	ShtCMR expert	ShtCMR non-expert	StdCMR	*p* (ShtCMR E vs. StdCMR)	*p* (ShtCMR non-E vs. StdCMR)	*p* (ShtCMR E vs. ShtCMR non-E)
LVEDV (mL)	135 ± 42	124 ± 40	137 ± 44	0.70	*< 0.0001*	*< 0.0001*
LVESV (mL)	56 ± 27	50 ± 30	61 ± 34	*0.001*	*< 0.0001*	*< 0.0001*
LVEDVi (mL/mq)	74 ± 19	68 ± 18	76 ± 20	0.425	*< 0.0001*	*< 0.0001*
LVESVi (mL/mq)	31 ± 12	27 ± 15	34 ± 16	*0.0005*	*< 0.0001*	*< 0.0001*
LVEF (%)	59 ± 11	61 ± 12	57 ± 11	*0.002*	*< 0.0001*	*< 0.0001*

*ShtCMR* short protocol, *StdCMR* standard protocol, *E* expert reader, *non-E* non-expert reader, *LVEDV* left ventricular end diastolic volume, *LVESV* left ventricular end systolic volume, *LVEDVi* left ventricular end diastolic volume index, *LVESVi* left ventricular end diastolic volume index, *LVEF* left ventricular ejection fraction

Significant differences were reported in italics

## Discussion

CMR has a crucial role in patients with a working diagnosis of MINOCA for addressing the correct diagnosis as recommended by the European and American guidelines [3, 5]. However, the StdCMR protocol requires a lengthy acquisition time and the administration of contrast medium and may not always be available or possible in emergency settings. A ShtCMR without contrast agent could be a possible alternative in some cases, provided it has the necessary reliability.

Our study compared the performance of a ShtCMR with that of its matched StdCMR in 179 cases of MINOCA patients. The ShtCMR considered lasts about 20 min and does not require contrast agent; it includes long-axis cine sequences for morphofunctional ventricular assessment and native T1, T2 mapping, and T2-STIR for tissue characterization in several cases.

The main finding of our study was the good agreement of the diagnoses performed with ShtCMR with the parallel StdCMR ones. It also evidenced that the confidence of the reader in the interpretation of the myocardial damage without use of contrast medium increased such agreement. Specifically, ShtCMR allowed reaching the same diagnosis as StdCMR in 85% of patients when interpreted by expert readers (rising from 66% for poor confidence to 99% for good, *p* = 0.0001) and 73% (*p* = 0.01) by non-expert ones (60% for poor vs 89% for good confidence, *p* = 0.0001).

These results might envision the implementation of a tailored approach which may avoid the use of contrast media in cases of good diagnostic confidence, thus allowing a shorter scanning time. In fact, based on the expert readers’ opinion, ShtCMR could have been sufficient to reach a diagnosis in 102/179 cases (57%) potentially leading to 41 h of scanning time saved (about 30%) at the cost of less than 1% incorrect diagnoses. Caution is however needed before considering any extension to clinical practice, since our results evidenced some issues related to an underestimation of the extent of the pathology and of volumes and overestimation of the ejection fraction.

Data on rigorously shortened CMR protocols is rare. Nadjiri et al [20] evaluated a shortened protocol based on T1 mapping and cine images in 160 individuals, finding that 70% of patients required contrast medium to complete the diagnostic process, in contrast to 34% (for the expert reader) and 43% (for the non-expert reader) of patients in our study population.

Hirschberg et al [15] evaluated the performance of a short CMR protocol without contrast agent in cardiomyopathy patients. As in our study, the scan time with ShtCMR was much shorter (23 vs. 48 min), and 92% of patients with suspected non-ischemic cardiomyopathies were diagnosed without contrast medium.

Even fewer data exist on short CMR protocols for MINOCA patients. Ferreira et al [21] showed that native T1 mapping can detect typical non-ischemic patterns with high sensitivity (90%) and specificity (88%) in patients with acute myocarditis and speculated that gadolinium-free CMR using cine and T1-mapping for tissue characterization may be possible in that setting. These findings are consistent with the revised Lake Louise criteria [12] which reported that a gadolinium-free protocol, combining T2-based CMR with T1 mapping, could be very appealing and effective in circumstances when contrast agents are not desirable. Vermes et al [13] evaluated 30 takotsubo patients and 34 controls, finding that a ShtCMR with mapping approaches techniques ensured high diagnosis accuracy, even if in Takotsubo patients there is usually no late enhancement [22].

The late gadolinium enhancement technique is important not only for diagnosis but also for prognosis in all heart diseases [23, 24]. With ShtCMR, LGE evaluation is not possible, and the extent of myocardial involvement is underestimated. This may be related to susceptibility to artifacts of the mapping technique especially in emergency patients with limited possibility of collaboration, which makes it harder to estimate the affected myocardial segments. However, the emergence and incorporation of mapping techniques in the evaluation of cardiac patients could make up for this deficit, both for their diagnostic and prognostic significance [25, 26].

Finally, in patients with MINOCA, assessing LV volume, wall motion, and ejection fraction is crucial for prognosis and therapy [3, 12, 27]. Bi-planar measurement is attractive because it only requires two views, reducing acquisition and processing time. Geometric assumptions limit biplane MRI. In our study, LV sizes were underestimated by 2–4 mL and LVEF was overestimated. Our findings are consistent with those of Huttin et al [28], who evaluated 100 patients with acute myocardial infarction and compared LV volume and EF with short-axis and long-axis techniques, finding a 63% reduction in acquisition/analysis time at the expense of a “systematic” underestimation of LVEDV (about 4 mL) and an overestimation of EF of about 5%.

Regarding the performance of readers with different experience, as one might expect, the less experienced readers had more difficulties with ShtCMR than the more experienced ones, but when the data is sub-analyzed for the different diagnoses, the difference was statistically significant only in the myocardial infarction group. We can speculate that for these patients, the diagnosis with ShtCMR without contrast medium may be hindered by the more difficult identification of the transmural distribution of the injury. In fact, the excellent specificity of LGE in providing a diagnosis is driven by the clear identification of the injured myocardial layer. Without contrast agent, edema can be subtler in relation to the severity of the damage or to the time delay from the symptom onset with unclear distribution pattern, and the presence of a microvascular obstruction may lead to a false absence of transmural distribution with subsequent misinterpretation of the injury as subendocardial/non ischemic. Additionally, the lack of short-axis cine images may affect the correct assessment of wall motion alteration. Nevertheless, in takotsubo cardiomyopathy, where the diagnosis is mainly guided by the identification of the typical contractile pattern, the presence of the apical ballooning is well explored with long axes cine images [27].

Our study has some limitations. Firstly, it is a retrospective study, even if the inclusion of consecutive patients from three different hospitals supports the robustness of the results. Second, we did not examine the contribution of the different sequences to the diagnosis, but made a diagnosis based on all sequences together without distinction. This approach was chosen because there is greater likelihood of reproducing the real approach used in clinical practice. Finally, the extent of the injury was assessed only qualitatively rather than quantitatively, so it is not possible to assess if differences are due to different degrees of severity.

In conclusion, ShtCMR was found to be a debatable alternative to the StdCMR in patients with MINOCA. Such a strategy could reduce time and resources without altering the patient’s diagnosis while also increasing the number of patients who can be scanned. However, this solution for being routinely acceptable requires the presence during scanning of an expert team able to quickly determine whether contrast administration is required. Alternatively, we may offer patients a first-stage ShtCMR and then, if necessary, add the contrast-enhanced part at a later scan.

## Abbreviations and acronyms

ACS: Acute coronary syndrome
cine-SSFP: Cine steady-state free precession
CMR: Cardiac magnetic resonance
GraSE: Gradient and spin echo sequence
IR-GRE: Inversion recovery gradient echo sequences
LGE: Late gadolinium enhancement
LVEDV: Left ventricular end diastolic volume
LVEF: Left ventricular ejection fraction
LVESV: Left ventricular end systolic volume
MINOCA: Myocardial infarction with non-obstructive coronary arteries
MOLLI: Modified Look-Locker inversion recovery
ShtCMR: Short non-contrast cardiac magnetic resonance
StdCMR: Standard comprehensive cardiac magnetic resonance
T2w-STIR: T2-weighted short tau inversion recovery

## Declarations

### Guarantor

The scientific guarantor of this publication is Prof. Paolo Fonio.

### Statistics and biometry

One of the authors has significant statistical expertise (Prof. Bergamasco).

### Methodology

• retrospective

• diagnostic study

• multicenter study

## References

[1] Bhatt DL, Lopes RD, Harrington RA (2022). Diagnosis and treatment of acute coronary syndromes: a review. JAMA.

[2] Palmisano A, Vignale D, Tadic M, et al (2022) Myocardial late contrast enhancement CT in troponin-positive acute chest pain syndrome. Radiology 302:545–553 10.1148/radiol.211288 34874200

[3] Collet J-P, Thiele H, Barbato E (2021). 2020 ESC Guidelines for the management of acute coronary syndromes in patients presenting without persistent ST-segment elevation. Eur Heart J.

[4] De Ferrari GM, Fox KAA, White JA (2014). Outcomes among non-ST-segment elevation acute coronary syndromes patients with no angiographically obstructive coronary artery disease: observations from 37,101 patients. Eur Heart J Acute Cardiovasc Care.

[5] Gulati M, Levy PD, Mukherjee D (2021). 2021 AHA/ACC/ASE/CHEST/SAEM/SCCT/SCMR guideline for the evaluation and diagnosis of chest pain: a report of the American College of Cardiology/American Heart Association Joint Committee on Clinical Practice Guidelines. Circulation.

[6] Pathik B, Raman B, Mohd Amin NH (2016). Troponin-positive chest pain with unobstructed coronary arteries: incremental diagnostic value of cardiovascular magnetic resonance imaging. Eur Heart J Cardiovasc Imaging.

[7] Dastidar AG, Baritussio A, De Garate E (2019). Prognostic role of CMR and conventional risk factors in myocardial infarction with nonobstructed coronary arteries. JACC Cardiovasc Imaging.

[8] Vágó H, Szabó L, Dohy Z (2020). Early cardiac magnetic resonance imaging in troponin-positive acute chest pain and non-obstructed coronary arteries. Heart.

[9] Sörensson P, Ekenbäck C, Lundin M (2021). Early comprehensive cardiovascular magnetic resonance imaging in patients with myocardial infarction with nonobstructive coronary arteries. JACC Cardiovasc Imaging.

[10] Monney PA, Sekhri N, Burchell T (2011). Acute myocarditis presenting as acute coronary syndrome: role of early cardiac magnetic resonance in its diagnosis. Heart.

[11] Dastidar AG, Harries I, Pontecorboli G (2019). Native T1 mapping to detect extent of acute and chronic myocardial infarction: comparison with late gadolinium enhancement technique. Int J Cardiovasc Imaging.

[12] Ferreira VM, Schulz-Menger J, Holmvang G (2018). Cardiovascular magnetic resonance in nonischemic myocardial inflammation: expert recommendations. J Am Coll Cardiol.

[13] Vermes E, Berradja N, Saab I (2020). Cardiac magnetic resonance for assessment of cardiac involvement in takotsubo syndrome: do we still need contrast administration?. Int J Cardiol.

[14] Baggiano A, Boldrini M, Martinez-Naharro A (2020). Noncontrast magnetic resonance for the diagnosis of cardiac amyloidosis. JACC Cardiovasc Imaging.

[15] Hirschberg K, Braun SM, Paul O, et al (2021) The diagnostic accuracy of truncated cardiovascular MR protocols for detecting non-ischemic cardiomyopathies. Int J Cardiovasc Imaging. 10.1007/s10554-021-02462-2 10.1007/s10554-021-02462-2 PMC11129993 34751885

[16] Kramer CM, Barkhausen J, Bucciarelli-Ducci C (2020). Standardized cardiovascular magnetic resonance imaging (CMR) protocols: 2020 update. J Cardiovasc Magn Reson.

[17] Palmisano A, Benedetti G, Faletti R (2020). Early T1 myocardial MRI mapping: value in detecting myocardial hyperemia in acute myocarditis. Radiology.

[18] Lawson MA, Blackwell GG, Davis ND (1996). Accuracy of biplane long-axis left ventricular volume determined by cine magnetic resonance imaging in patients with regional and global dysfunction. Am J Cardiol.

[19] Leiner T, Bogaert J, Friedrich MG (2020). SCMR Position Paper (2020) on clinical indications for cardiovascular magnetic resonance. J Cardiovasc Magn Reson.

[20] Nadjiri J, Zaschka A-L, Straeter AS (2019). Evaluation of a shortened cardiac MRI protocol for left ventricular examinations: diagnostic performance of T1-mapping and myocardial function analysis. BMC Med Imaging.

[21] Ferreira VM, Piechnik SK, Dall’Armellina E (2014). Native T1-mapping detects the location, extent and patterns of acute myocarditis without the need for gadolinium contrast agents. J Cardiovasc Magn Reson.

[22] Gatti M, Carisio A, D’Angelo T (2020). Cardiovascular magnetic resonance in myocardial infarction with non-obstructive coronary arteries patients: a review. World J Cardiol.

[23] Dang Y, Hou Y (2021). The prognostic value of late gadolinium enhancement in heart diseases: an umbrella review of meta-analyses of observational studies. Eur Radiol.

[24] Georgiopoulos G, Figliozzi S, Sanguineti F (2021). Prognostic impact of late gadolinium enhancement by cardiovascular magnetic resonance in myocarditis: a systematic review and meta-analysis. Circ Cardiovasc Imaging.

[25] Yang M-X, Luo H-B, Liu J-K, et al (2022) Prognostic value of non-contrast myocardial T1 mapping in cardiovascular diseases: a systematic review and meta-analysis. Heart Fail Rev 27:1899–1909 10.1007/s10741-021-10191-w 35064397

[26] Snel GJH, van den Boomen M, Hernandez LM (2020). Cardiovascular magnetic resonance native T2 and T2* quantitative values for cardiomyopathies and heart transplantations: a systematic review and meta-analysis. J Cardiovasc Magn Reson.

[27] Ghadri J-R, Wittstein IS, Prasad A (2018). International expert consensus document on takotsubo syndrome (Part I): clinical characteristics, diagnostic criteria, and pathophysiology. Eur Heart J.

[28] Huttin O, Petit M-A, Bozec E (2015). Assessment of left ventricular ejection fraction calculation on long-axis views from cardiac magnetic resonance imaging in patients with acute myocardial infarction. Medicine (Baltimore).

